# Real-world retrospective observational study exploring the effectiveness and safety of antifibrotics in idiopathic pulmonary fibrosis

**DOI:** 10.1136/bmjresp-2020-000782

**Published:** 2021-03-26

**Authors:** William Alexander Wright, Louise E Crowley, Dhruv Parekh, Anjali Crawshaw, Davinder P Dosanjh, Peter Nightingale, David R Thickett

**Affiliations:** 1 School of Medical and Dental Sciences, Institute of Inflammation and Ageing, University of Birmingham, Birmingham, West Midlands, UK; 2 Department of Respiratory Medicine, University Hospitals Birmingham NHS Foundation Trust, Birmingham, Birmingham, UK; 3 Institute of Inflammation and Ageing, University of Birmingham, Birmingham, West Midlands, UK

**Keywords:** interstitial fibrosis

## Abstract

**Background:**

Pirfenidone and nintedanib are the only disease-modifying treatments available for idiopathic pulmonary fibrosis (IPF). Our aim was to test their effectiveness and safety in clinical practice.

**Methods:**

This is a single-centre retrospective observational study undertaken at a specialised interstitial lung disease centre in England. Data including progression-free survival (PFS), mortality and drug tolerability were compared between patients with IPF on antifibrotic therapies and an untreated control group who had a forced vital capacity percentage (FVC %) predicted within the licensed antifibrotic treatment range.

**Results:**

104 patients received antifibrotic therapies and 64 control patients were identified. PFS at 6 months was significantly greater in the antifibrotic group (75.0%) compared with the control group (56.3%) (p=0.012). PFS was not significant at 12 or 18 months when comparing the antifibrotic group with the control group. The 12-month post-treatment mean decline in FVC % predicted (−4.6±6.2%) was significantly less than the 12-month pretreatment decline (−10.4±11.8%) (p=0.039). The 12-month mortality rate was not significantly different between the antifibrotic group (25.3%) and the control group (35.5%) (p=0.132). Baseline Body Mass Index of≤25, baseline diffusion capacity for carbon monoxide percentage predicted of ≤35 and antifibrotic discontinuation within 3 months were independent predictors of 12-month mortality. Antifibrotic discontinuation was significantly higher by 3 and 6 months for patients on pirfenidone than those on nintedanib (p=0.006 and p=0.044, respectively). Discontinuation at 12 months was not significantly different (p=0.381).

**Conclusions:**

This real-world study revealed that antifibrotics are having promising effects on PFS, lung function and mortality. These findings may favour commencement of nintedanib as first-line antifibrotic therapy, given the lower rates of early treatment discontinuation, although further studies are required to investigate this.

**Key messages**

- Is the efficacy of antifibrotics shown in clinical trials being translated to everyday clinical practice?

- This real-world study revealed that antifibrotics are having promising effects on progression-free survival, lung function and mortality, and nintedanib appears better tolerated than pirfenidone in the early stages of antifibrotic therapy.

- The lower rates of treatment discontinuation for patients treated with nintedanib may have positive outcomes on mortality.

## Introduction

Idiopathic pulmonary fibrosis (IPF), the the most common form of interstitial lung disease (ILD) worldwide, is a devastating condition, characterised by progressive fibrosis, dyspnoea and ultimately death within a median time of 3–5 years.^1^ The pharmacological management of IPF has evolved significantly over the last 10 years following the results of the PANTHER trial^2^ with a shift from anti-inflammatory to antifibrotic therapies.

Nintedanib and pirfenidone are the only antifibrotic therapies licensed and approved for use in clinical practice in Europe. Multinational randomised control trials (RCTs)^3–5^ have shown that these therapies are able to potentially slow disease progression represented by slowed forced vital capacity (FVC) decline.

However, these RCTs to date were not powered to assess the effect of antifibrotic therapies on mortality. Pooled analyses and meta-analyses have been undertaken to assess this. Nathan *et al*^6^ and Aravena *et al*^7^ conducted pooled analyses and meta-analyses of pirfenidone treatment, which both revealed significantly reduced mortality at 52 weeks. Richeldi *et al*^8^ carried out a pooled analysis and meta-analysis of the INPULSIS and TOMORROW trials of nintedanib for IPF. The pooled analysis revealed a significant reduction in on-treatment mortality, but none of the mortality outcomes were significant in the meta-analysis. Due to the RCTs protocols’ strict inclusion criteria of only mild to moderate disease severity and excluding multiple comorbidities, fewer deaths would be expected than in routine clinical practice.

Although RCTs are a robust method of assessing efficacy and safety, real-world studies provide insight into the ongoing effectiveness of medications in everyday clinical practice. The results are often more generalisable due to not having the limitations of eligibility criteria.

Many real-world studies have been carried out since the licensing of these medications, which have shown the disease-stabilising effect of these drugs through sequential FVC measurements.^9–14^ These studies have also assessed antifibrotic tolerability with the discontinuation rate for pirfenidone approximately 29% and for nintedanib between 11% and 19%.^9 10 13 14^

The aim of this study was to assess the efficacy and safety of the use of antifibrotic therapies in the management of IPF in everyday clinical practice in the UK. This study is novel as it includes a control group of patients not receiving antifibrotic therapies, allowing comparisons to be made and the effectiveness and tolerability of antifibrotics to be elicited.

## Methods

### Project design

This is a single-centre retrospective observational study undertaken at the Queen Elizabeth Hospital, Birmingham (QEHB), which is a National Health Service England specialist commissioned ILD service.

### Patients and public involvement

Patients were not involved in the design, conduct or dissemination of the study.

### Data collection

Patients with IPF were identified through review of monthly ILD multidisciplinary team (MDT) meeting records from October 2011 to October 2017 at QEHB (records from 63 meetings were available for review). Patients with definite and probable usual interstitial pneumonia (UIP) with no identifiable cause were diagnosed with IPF, in accordance with American Respiratory Society, European Respiratory Socierty Japanese Respiratory Society and Latin American Thoracic Association (ATS/ERS/JRS/ALAT) guidelines.^15^ Patients with IPF were eligible for antifibrotic treatment, providing their forced vital capacity percentage (FVC %) predicted was between 50% and 80%.^16 17^ Following the approval of pirfenidone by the Medicines and Healthcare Regulatory Authority (MHRA) and National Institute for Health and Care Excellence, QEHB was set up as an NHS England specialty commission ILD clinic to prescribe antifibrotics, one of four such clinics in the West Midlands region (population of five million). Initially, pirfenidone was the only drug available until May 2015 when nintedanib was also approved. Patients subsequent to May 2015 were offered a free choice after being given information about the efficacy and side effects of the medications. There are no specific contraindications to either antifibrotic.

Patients included in this study were commenced on pirfenidone between August 2013 and September 2018 and nintedanib between May 2015 and July 2018. Control patients were identified as patients with IPF who had never received antifibrotics but met the lung function criteria of FVC % predicted of 50%–80%. Most included patients were reviewed at the ILD MDT after the results of the PANTHER study^2^ and practice at QEHB was not to use anti-inflammatory therapies following this study.

For the control group, the date of the first lung function test to have an FVC between 50% and 80% predicted was used as the ‘reference point’, namely, the point where an antifibrotic could have been prescribed and the date the sequential data were taken from. The date of the clinic when the antifibrotic was first prescribed was used as the reference point for the antifibrotic group. Data were collected retrospectively using electronic patient medical records and included mortality, lung function and antifibrotic discontinuation. Outside of a clinical trial, lung function measurements are not standardised in IPF treatment pathways, particularly in patients diagnosed in other hospitals subsequently referred to specialist centres. Nevertheless, we analysed all of the available lung function for the antifibrotic group before and after commencing treatment.

### Statistical analysis

IBM SPSS v16 Statistics was used to carry out analysis of the data. Continuous variables are presented as means with SD for parametric data and medians with IQR for non-parametric data. Data were tested for normality using the Shapiro-Wilk test. Pearson’s χ^2^ test was used when comparing categorical variables between groups and the independent samples t-test when comparing continuous parametric variables. The Mann-Whitney U test was used for comparing continuous non-parametric variables. A paired samples t-test was used when comparing pretreatment and post-treatment 12-month change in FVC % predicted. Survival curves were formed using Kaplan-Meier analysis, and the groups were compared using the log-rank test (Mantel-Cox). For the univariate analysis of predictors of 12-month mortality and drug discontinuation, continuous variables were converted to binary outcomes. Pearson’s χ^2^ test was then used to analyse these variables for significance. For the multivariate analysis, forward logistic regression with a CI of 95% was performed.

## Results

A total of 104 patients with IPF who had been commenced on antifibrotics at any point were included in the analysis. Seventy-one control patients who had not received antifibrotics but met the lung function eligibility criteria were identified. Seven patients were excluded, either for receiving an antifibrotic just before the end of the monitoring period (n=5), for having previously received an antifibrotic elsewhere (n=1) or for having had only one FVC recording within eligibility range, which on further review was noted to be an inaccurate result (n=1). This resulted in a final control group of 64 patients who were included in the analyses (online supplemental figure 1). Reasons for the control patients never having started antifibrotics include patients only meeting FVC criteria in the pre-antifibrotic era (n=12), patients declining following discussion of potential side effects or as they did not feel limited by the disease (n=34), relative contraindications to antifibrotic therapy (n=16) and death prior to commencing treatment (n=2). Twelve months of follow-up data were collected for all 168 patients and 18-month data were available for 163 patients (100 antifibrotic treated and 63 control patients).

10.1136/bmjresp-2020-000782.supp1 Supplementary data

### Baseline characteristics

A total of 104 patients on antifibrotics and 64 control patients were included in the analysis. Baseline characteristics were mostly comparable between the antifibrotic and control groups (table 1). However, the control patients were significantly older with a median age of 78 years as compared with 72 years for the antifibrotic group (p=0.004) and had a significantly higher median baseline FVC of 71% predicted compared with 67% predicted for the antifibrotic group (p=0.015). Significantly more patients in the antifibrotic group were referred from external hospitals and had previously received corticosteroid therapy than the control group (54.8% vs 32.8% (p=0.006) and 19.2% vs 6.3% (p=0.02), respectively). Of the 104 patients prescribed antifibrotics, 62 patients were first commenced on pirfenidone and 42 on nintedanib. Fourteen (13.5%) of these patients switched from one of the antifibrotics to the other. Baseline characteristics for patients initiated on the two medications are presented in online supplemental table 1.

**Table 1:** Baseline characteristics of the antifibrotic group compared with the control group

Characteristics	Antifibrotic (n=104)	Control (n=64)	P value
Male sex, n (%)	79 (76.0)	49 (76.6)	0.929
Age (years), median (IQR)	**72** (**12**)	**78** (**12**)	**<0.001**
Smoking status, n (%)			0.354
Never	33 (32.4)	20 (31.3)	
Ex-smoker	67 (65.7)	40 (62.5)	
Current smoker	2 (2.0)	4 (6.25)	
Smoking pack-years, median (IQR)	11 (25)	13 (35)	0.470
Weight (kg), mean (SD)	79.9 (17.5)	76.9 (14.5)	0.253
BMI, median (IQR)	28.48 (5.48)	26.85 (5.77)	0.122
External referral, n (%)	**57** (**54.8**)	**21** (**32.8**)	**0.006**
Oxygen therapy (%)			
LTOT	8 (7.7)	5 (7.8)	0.977
Ambulatory	15 (14.4)	5 (7.8)	0.199
Previous triple therapy, n (%)	5 (4.8)	2 (3.1)	0.596
Previous corticosteroid therapy, n (%)	**20** (**19.2**)	**4** (**6.3**)	**0.020**
Histological diagnosis, n (%)	16 (15.4)	5 (7.8)	0.150
FVC (L), mean (SD)	2.26 (0.51)	2.31 (0.53)	0.520
FVC % predicted, median (IQR)	**67.0** (**14.5**)	**71.0** (**11.2**)	**0.015**
DLco (mmol/min/kPa), median (IQR)	3.36 (1.48)	3.11 (1.63)	0.343
DLco % predicted, mean (SD)	43.0 (12.6)	42.1 (14.3)	0.694
Kco (mmol/min/kPa/L), mean (SD)	1.07 (0.31)	0.99 (0.32)	0.120
Kco % predicted, mean (SD)	80.4 (23.7)	77.4 (23.2)	0.447
SpO_2_ (%), median (IQR)	94 (4)	95 (6)	0.597
Emphysema on HRCT, n (%)	14 (13.5)	13 (20.3)	0.240

BMI, Body Mass Index; DLco, diffusion capacity for carbon monoxide; DLCO, diffusion capacity for carbon monoxide percentage; FVC, forced vital capacity percentage; FVC, forced vital capacity; HRCT, High resolution Computed tomography scan; IQR, interquartile range; Kco, carbon monoxide transfer coefficient; SpO_2_, pulse oximetry.

### Mortality

**Figure 1:**
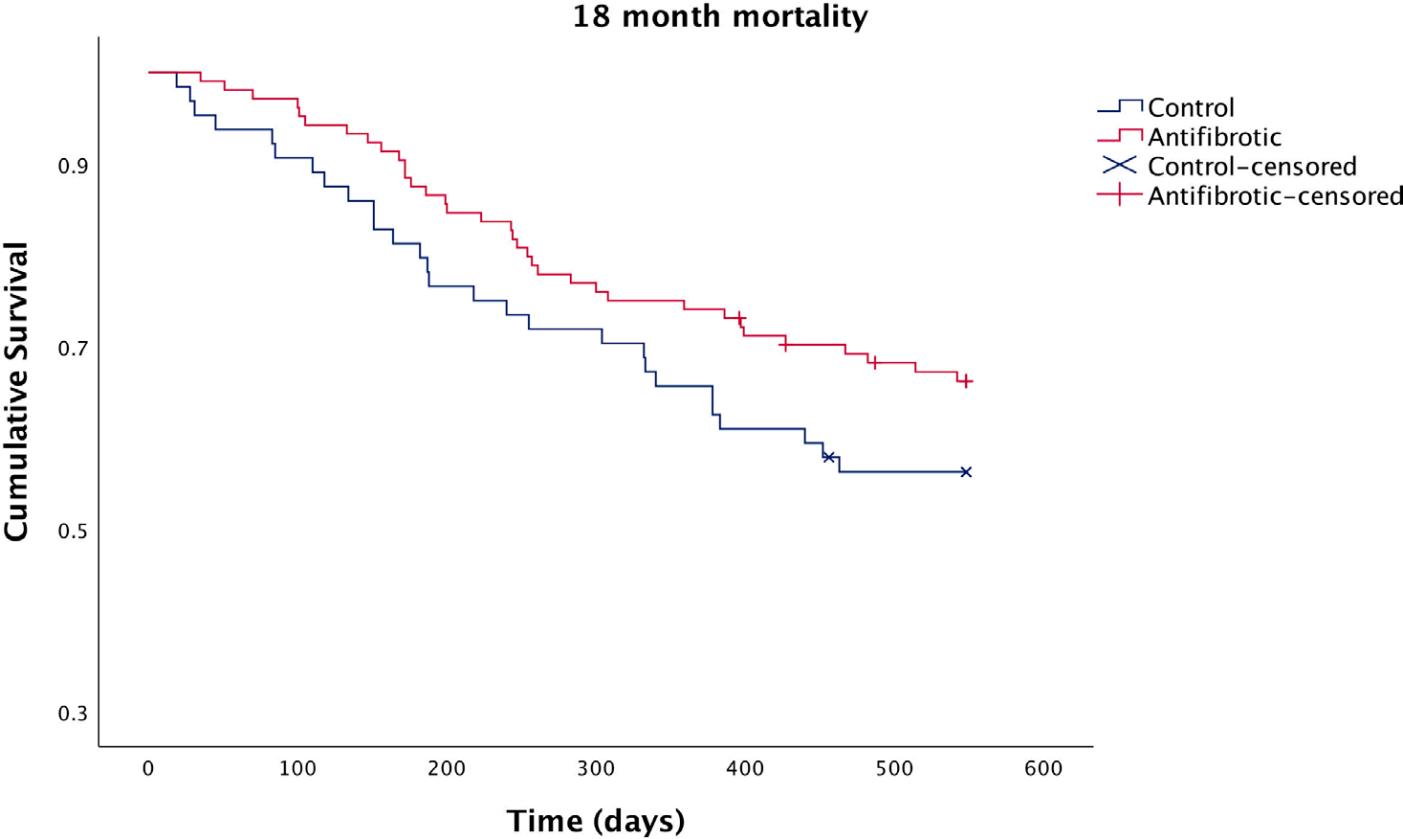
Kaplan-Meier curve comparing 18-month survival in patients receiving antifibrotics and in control patients. Censored: patients without 18 months of follow-up at the time of analysis.

Mortality is summarised in figure 1. Univariate regression analysis showed that baseline Body Mass Index (BMI) of ≤25, baseline predicted diffusion capacity for carbon monoxide (DLCO)≤35% and baseline supplementary oxygen (ambulatory or LTOT) were predictors of 12-month mortality. On multivariate analysis, only baseline BMI of ≤25 (OR 3.3, 95% CI 1.44 to 7.59; p=0.005) and baseline predicted DLco of ≤35% (OR 3.3, 95% CI 1.44 to 7.59; p=0.005) were independent predictors of 12-month mortality. These parameters were comparable between the control and antifibrotic groups. Age of ≥75 years was not found to be an independent risk factor for 12-month mortality. Full data from the multivariate analysis are presented in the online supplementary material.

On an intention-to-treat basis, after adjustment of independent predictors identified on multivariate analyses previously, mortality rates of 6, 12 and 18 months were lower in the antifibrotic group as compared with the control group, although these were not statistically significant (online supplemental table 2). Twewlve-month mortality was higher than reported in clinical trials (26% in the antifibrotic group vs 34.4% in the control group) (table 2). There were no significant mortality differences when comparing the pirfenidone and nintedanib groups.

**Table 2:** Mortality and PFS in antifibrotic and control groups for patients with 6, 12 and 18 months of follow-up

	Antifibrotic (n=104)	Control (n=64)	P value
6-month mortality (%)	13 (12.5)	13 (20.3)	0.174
12-month mortality (%)	27 (26.0)	22 (34.4)	0.244
18-month mortality (%)	34 (34.0)*	28 (44.4)†	0.181
PFS of 6 months (%)	78 (75.0)	36 (56.3)	0.012
PFS of 12 months (%)	53 (51.0)	22 (34.4)	0.036
PFS of 18 months (%)	37 (37.0)*	16 (25.4)†	0.124

*n=100 for antifibrotic-treated patients with 18-month follow up.

†n=63 for control patients with 18-month follow up.

PFS, progression-free survival.

### Progression-free survival (PFS)

PFS was defined as those who had lived and experienced a less than 10% decline in FVC. Multivariate analysis was performed to identify predictors of PFS of 6, 12 and 18 months. This analysis revealed that age of ≥75 years was an independent predictor (OR 2.12, 95% CI 1.08 to 4.14; p=0.029) of 12-month PFS.

PFS at 6 months was significantly improved in the antifibrotic group (75.0%) compared with the control group (56.3%) (p=0.012). This improvement was not maintained at 12 months (having adjusted for age of ≥75 years) or 18 months. No adjustment for risk factors was applied for 6 and 18 months as there were no independent predictors identified in the multivariate analysis.

### Lung function

**Figure 2:**
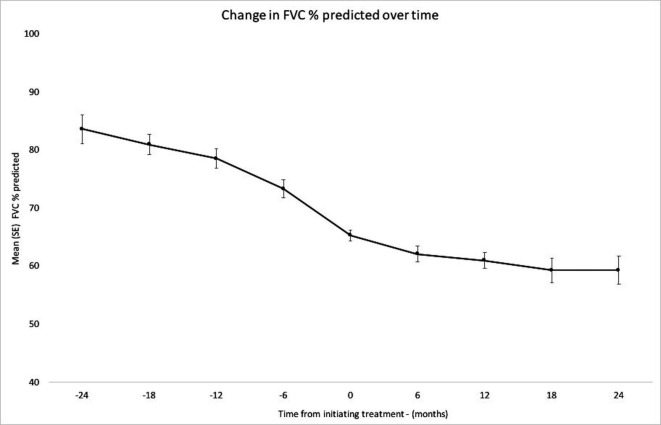
Decline in mean FVC % predicted from 24 months before and 24 months after commencing antifibrotic therapies. 0 is the point at which antifibrotics were started. SE of each mean is also presented. Number of patients with an FVC measurement at each time point: −24 (23), −18 (28), −12 (48), −6 (42), 0 (103), 6 (56), 12 (55), 18 (40), and 24 (24). FVC, forced vital capacity; FVC %, forced vital capacity percentage.

Change in lung function is summarised in figure 2. The post-treatment 12-month decline in mean FVC % predicted (4.6%±6.2%) was significantly less than the pretreatment 12-month decline (10.4%±11.8%) (p=0.039). This result was based on matched data from 26 patients who had a complete set of the required FVC measurements. The same effect was seen in patients without paired data (15.1%±9.5% for n=22 patients with FVC data 12 months prior to treatment initiation and 5.5%±8.5% for n=28 patients with FVC data 12 months after treatment initiation, p<0.001).

### Side effects and discontinuation

The most common side effect seen in patients who were treated with pirfenidone was nausea (36.8% of patients). Other common side effects were appetite loss (20.6%), dyspepsia (17.6%) and lethargy (16.2%). Patients (11.8%) had a photosensitivity reaction despite being advised to use sun cream. Diarrhoea was the predominant side effect for the nintedanib-treated patients, with 62% of patients experiencing it. Nausea (28%) and appetite loss (24%) were other common side effects.

**Figure 3:**
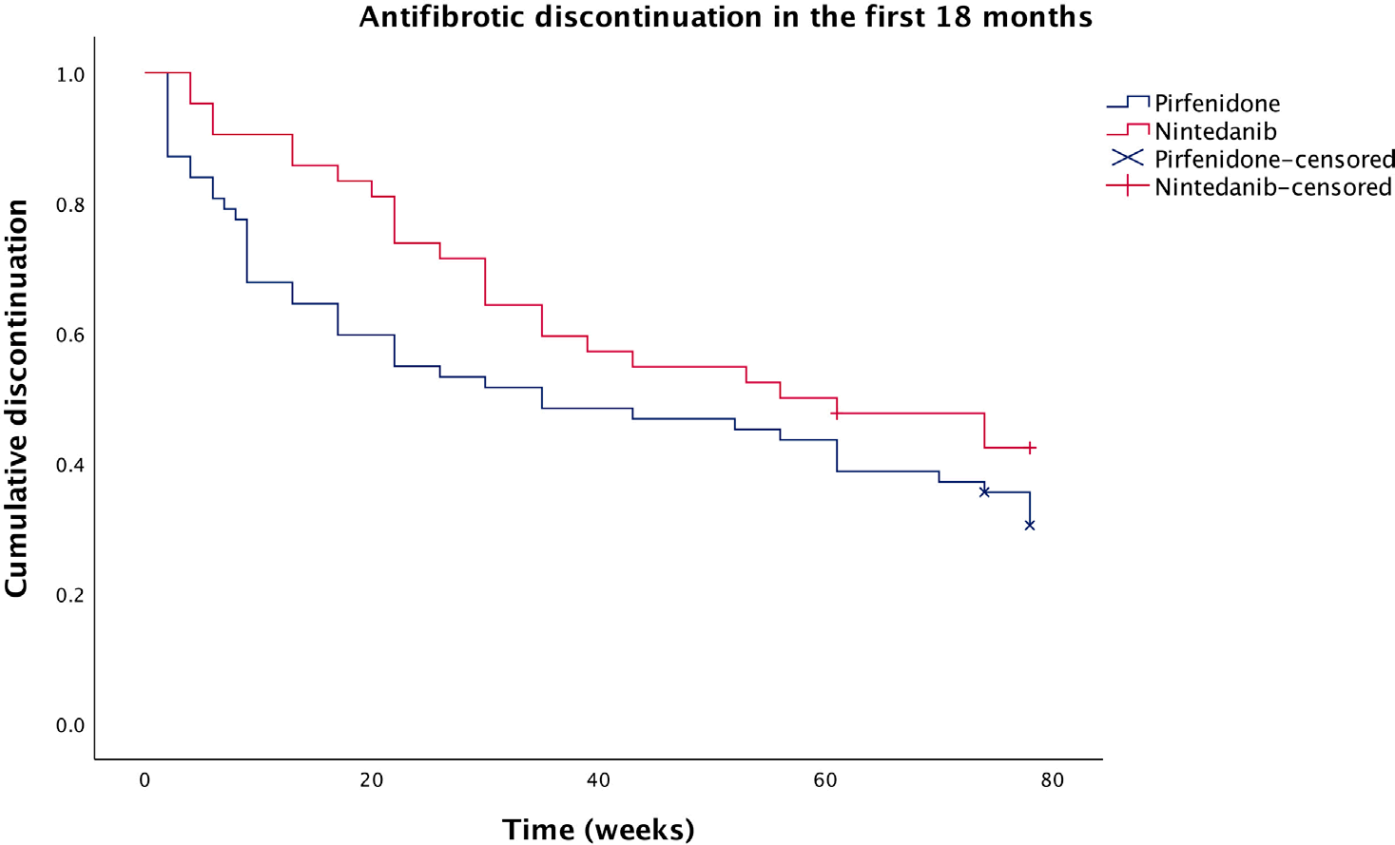
Kaplan-Meier curve comparing antifibrotic discontinuation over the first 18 months of treatment between patients receiving nintedanib and pirfenidone. Censored: patients without 18 months of follow-up at the time of analysis.

For the discontinuation analysis, patients who had taken both antifibrotics were included, but only their first antifibrotic trial. Therefore, switching to their second antifibrotic counted as a discontinuation. Significantly more patients had discontinued pirfenidone (n=18, 29.0%) than nintedanib (n=3, 7.1%) by 3 months (p=0.006). Similarly, significantly more patients had discontinued pirfenidone (n=25, 40.3%) than nintedanib (n=9, 21.4%) by 6 months (p=0.044). However, the difference was no longer significant at 12 months (n=29 (46.8%) for pirfenidone, n=16 (38.1%) for nintedanib; p=0.381) or 18 months (n=35 (58.3%) for pirfenidone, n=21 (52.5%) for nintedanib; p=0.564). The discontinuation comparison is summarised in figure 3.

The effect of antifibrotic treatment on mortality was further evaluated by excluding from the analysis patients who discontinued treatment within 3 and 6 months. Twelve-month mortality in the antifibrotic group, excluding patients who discontinued treatment within 6 months (n=70), was significantly lower than the control group (n=11 (15.7%) vs n=22 (34.4%), p=0.012, respectively). This was also true for 18-month mortality (n=17 (25.0%) vs n=28 (44.4%), p=0.019).

Multivariate analysis of the antifibrotic group revealed discontinuation of an antifibrotic within 3 months as a significant predictor of 12-month mortality (OR 6.26, 95% CI 1.54 to 25.52; p=0.010). This analysis also showed baseline BMI of ≤25 (OR 14.40, 95% CI 3.68 to 56.34; p=<0.001) and baseline platelet count of ≤200 (OR 4.09, 95% CI 1.13 to 14.77; p=0.031) were independent predictors of 12-month mortality.

Univariate and multivariate analyses were also performed to identify predictors of antifibrotic discontinuation. Univariate analysis suggested age of ≥75 years, female sex and baseline BMI of ≤25 were predictors of discontinuation by 12 months. On multivariate analysis, age of ≥75 years (OR 3.17, 95% CI 1.16 to 8.68; p=0.025), female sex (OR 4.57, 95% CI 1.40 to 14.88; p=0.012) and baseline BMI of ≤25 (OR 3.74, 95% CI 1.22 to 11.53; p=0.021) were also found to be independent predictors of discontinuation by 12 months.

## Discussion

This study provides real-world evidence of improved PFS at 6 months and reduced decline in FVC over time in patients with IPF treated with antifibrotic therapies. Although motality of 6, 12 and 18 months appeared lower in the antifibrotic group, this was not significant. However, exclusion of patients who discontinued treatment within 6 months from the analysis resulted in significantly improved mortality in the antifibrotic group. Patients appeared to tolerate nintedanib better than pirfenidone for the first 3–6 months of treatment. Continued treatment in the first 3 to 6 months of antifibrotic therapy was associated with significantly improved 12 month mortality. Multivariate analysis of the entire cohort identified BMI of ≤25 and DLco of ≤35% predicted as risk factors for mortality. Baseline BMI of ≤25, age of ≥75 years and female sex were found to be risk factors for antifibrotic discontinuation secondary to both adverse drug reactions and disease progression.

The promising effects of antifibrotics on PFS and FVC decline, but absence of significant effect on mortality, are consistent with the results of the clinical trials.^3–5^ However, the mortality rates in this study are higher than those seen in RCTs, which we speculate are secondary to more lenient inclusion criteria for antifibrotic therapies in everyday clinical practice. For example, the antifibrotic group appeared to be of older age and to have worse DLco than the pirfenidone arm of the CAPACITY trial.^3^ Patients in real-life clinical practice are likely to have more comorbidities as trials such as the ASCEND trial excluded patients likely to die within 2 years.^4^ The intolerance of antifibrotics is high when compared with the clinical trials. This highlights the importance of real-world data to inform clinical decision making as the availability of counselling and resources may be different in these settings when compared with trial conditions.

The discontinuation rates at 3 and 6 months were significantly better with nintedanib, which we have shown could have beneficial effects on survival. These findings may favour commencement of nintedanib as first-line antifibrotic therapy. However, this appears to differ from previous work that showed discontinuation rates were comparable between nintedanib and pirfenidone,^18 19^ and so further studies are required to investigate this. Discontinuation rates here were also higher than a recent US pulmonary fibrosis registry-based study where it was only around 11%, which may be related to different healthcare settings.^18^

There are cases where pirfenidone would be the preferred therapy over nintedanib, such as due to comorbidities or pharmacological interactions. This study shows that pirfenidone is less well tolerated at all time points, but more so in the first 6 months as following this, the Kaplan-Meier curve flattens out. Of the patients who stopped pirfenidone by 12 months, 86% had stopped within 6 months. This suggests that patients who can tolerate the early phase of therapy are more likely to continue long term with discontinuation rates at 12 months not significantly different from nintedanib. Nausea was the most frequent side effect in patients on pirfenidone, which can be managed through concomitant prescription of an antiemetic. Loperamide was often coprescribed with nintedanib and found to be effective. However, the same effectiveness may not have been seen with pirfenidone as often it is a combination of side effects with general malaise that leads to discontinuation.

The side-effect profile seen here is like those in previous trials. In a pooled analysis^9^ of the TOMORROW and INPULSIS trials of nintedanib, 61.5% and 24.3% of patients experienced diarrhoea and nausea, respectively, similar to here. For pirfenidone, rash and diarrhoea were less frequently reported than in a pooled analysis of the CAPACITY and ASCEND trials,^20^ although a very similar proportion of patients suffered from nausea. The much lower reporting of photosensitive rash may be attributed to the effectiveness of counselling patients regarding the use of sunblock. For both medications, appetite loss was more common in our study, which is most likely due to the older, multimorbid patient group.

DLco has previously also been shown to be a predictor of mortality.^21–23^ This is also true for baseline BMI in both the preantifibrotic and postantifibrotic eras.^24 25^ Decline in BMI has also been shown to be a risk factor for increased mortality.^26 27^ Our results are surprising as it appears patients with a normal BMI are at an increased risk of dying. However, this finding, alongside DLco as a predictor of mortality, is consistent with Fang *et al*’s real-world pirfenidone study.^28^ We also noted that baseline BMI was a predictor of antifibrotic discontinuation, which has been reported previously,^29^ with Kato *et al*^30^ finding that low BMI increases the risk of experiencing nintedanib-associated gastrointestinal side effects.

Age^31 32^ and female sex^29 31^ have also been shown to be risk factors for discontinuation previously. The results of the multivariate analysis suggest that there are demographic factors that influence discontinuation that need further study.

### Limitations

The limitations of the study are related largely to those encountered in all real-world retrospective analyses. The infrequency of lung function being performed in everyday clinical practice led to missing FVC data. FVC data imputation was not carried out for patients who died. This was addressed through the use of PFS as an outcome when comparing the two groups. There are also limitations with the control group used. This control group was formed retrospectively. Some of these patients were not started on antifibrotics as they had stable disease, potentially reducing the positive effect of the antifibrotics on PFS. There were some differences in the baseline characteristics of the two groups, but these variables were not found to independently influence mortality. The overall number of patients in this study is relatively small and is particularly limited by the size of the control group (n=64). We also acknowledge that this is a single centre study, which limits the generalisation of the data to the diversity of NHS ILD centres in the UK.

## Conclusions

This retrospective study shows that in real-world clinical practice antifibrotics are having promising effects on PFS, lung function and mortality. Both antifibrotics have been shown to have acceptable safety profiles, although the data suggest nintedanib is better tolerated, which could have an influence on survival. The identification of clinical predictors of mortality and discontinuation may lead to a more stratified approach to the treatment of patients with IPF in the future.
