# Supplementary material for: Real-world retrospective observational study exploring the effectiveness and safety of antifibrotics in idiopathic pulmonary fibrosis
Source: BMJ Open Respir Res. 2021 Mar 26;8(1):e000782. doi: 10.1136/bmjresp-2020-000782 (PMC8006845; doi:10.1136/bmjresp-2020-000782)
Supplement: Supplementary data [file bmjresp-2020-000782supp001.pdf]

A real world retrospective observational study exploring the effectiveness and safety of antifibrotics in idiopathic pulmonary fibrosis

Wright WA et al.

Online Supplementary Material

Supplementary Figure 1: Consort diagram showing the process of patient inclusion and exclusion from analyses in the antifibrotic and control groups. 'Both antifibrotics' include patients who switched from one antifibrotic to another. ILD – interstitial lung disease, MDT – multi-disciplinary team, IPF – idiopathic pulmonary fibrosis, FVC – forced vital capacity.

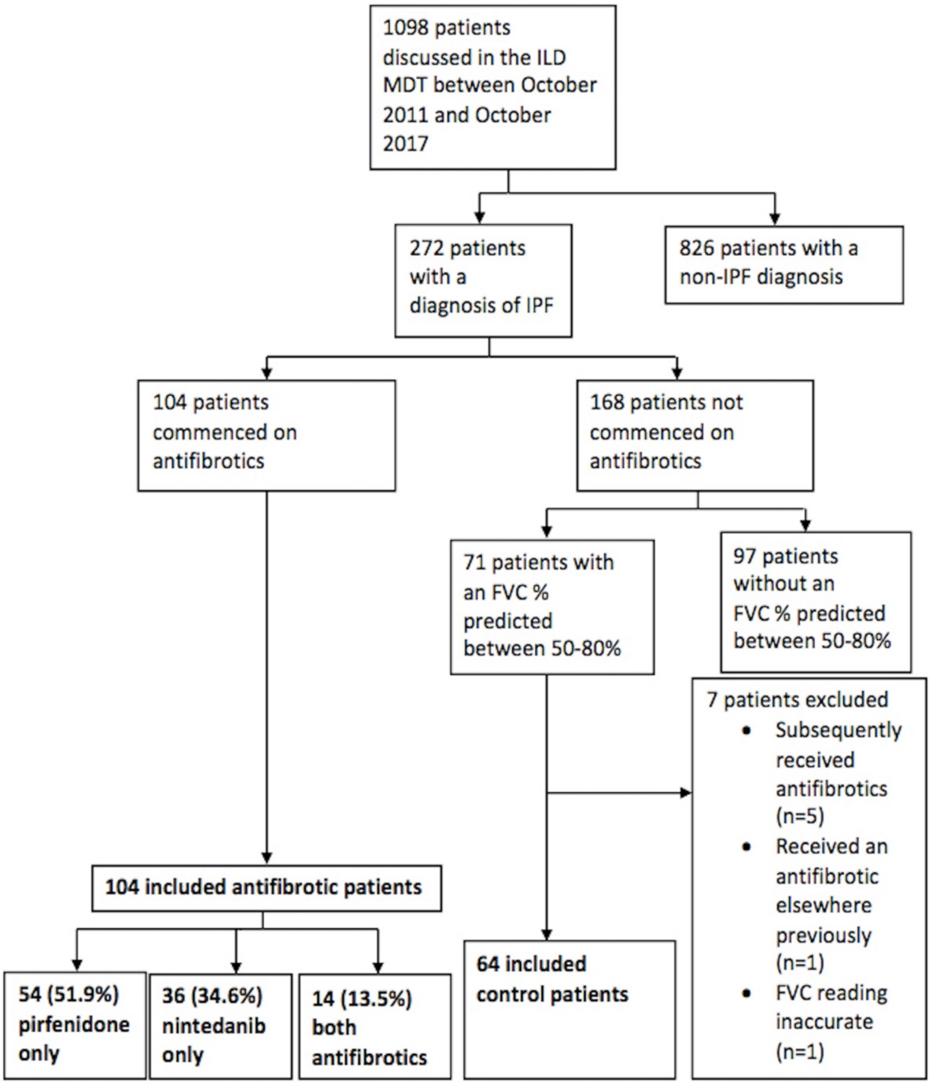

Supplementary Table 1: Baseline characteristics of the patients who commenced pirfenidone first compared to those who commenced nintedanib first. IQR – interquartile range, BMI – body mass index, FVC - Forced vital capacity, DLco – diffusion capacity for carbon monoxide, Kco -carbon monoxide transfer coefficient, SpO_2 – pulse oximetry.

**Characteristic**		**Pirfenidone (n=62)**	**Nintedanib (n=42)**	**P value**
Male sex – no. (%)		45 (72.6)	34 (81.0)	0.327
Age – mean (SD)		69.9 (8.7)	72.6 (7.7)	0.108
Smoking status (%)				
	Never	19 (31.1)	14 (34.1)	
	Ex-smoker	40 (65.6)	27 (65.9)	
	Current smoker	2 (3.2)	0 (0.0)	
Smoking pack years (IQR)		17 (25)	8 (25)	0.381
Weight – Kg (SD)		81.8 (18.4)	77.3 (12.0)	0.197
BMI (IQR)		28.70 (6.27)	28.09 (4.70)	0.113
External referral (%)		35 (56.5)	22 (52.4)	0.682
Oxygen therapy (%)				
	LTOT	7 (11.3)	1 (2.4)	0.094
	Ambulatory	8 (12.9)	7 (16.7)	0.592
Previous triple therapy (%)		5 (8.1)	0 (0.0)	0.059
**Previous corticosteroid therapy (%)**		**17 (27.4)**	**3 (7.1)**	**0.010**
Histological diagnosis (%)		8 (12.9)	8 (19.0)	0.394
FVC – L (SD)		2.25 (0.50)	2.27 (0.53)	0.860
FVC % predicted (SD)		65.8 (9.3)	64.7 (8.5)	0.532
DLco – mmol/min/kPa (IQR)		3.21 (1.28)	3.48 (1.81)	0.215
DLco % predicted (SD)		41.5 (13.0)	45.2 (11.9)	0.167
Kco – mmol/min/kPa/L (SD)		1.04 (0.32)	1.13 (0.31)	0.219
Kco % predicted (SD)		77.5 (23.4)	84.7 (23.7)	0.153
SpO_2 – % (IQR)		94 (5)	94 (4)	0.582
Emphysema on HRCT (%)		7	7	0.431

Supplementary Table 2: Effect of antifibrotic treatment on mortality at 6, 12 and 18 months with adjustment for independent predictors of mortality for each time point. Adjustment for baseline BMI □25 baseline and predicted DLco □35% at 12 and 18 months. Adjustment for DLco □35%, Baseline eGFR ≤50, Baseline neutrophil : lymphocyte ratio ≥5 at 6 months.

	**Odds ratio (CI)**	**P Value**
Antifibrotic treatment 6-month mortality	0.44 (0.14-1.31)	0.140
Antifibrotic treatment 12-month mortality	0.56 (0.24-1.30)	0.178
Antifibrotic treatment 18-month mortality	0.59 (0.27-1.32)	0.199

Univariate and multivariate analysis for 12-month mortality

Supplementary Table 3: Univariate analysis for 12-month mortality. Supplementary oxygen is patients on either LTOT or ambulatory oxygen therapy.

**Variable**	**N**	**Odds ratio (CI)**	**Likelihood ratio**	**P value**
Age ≥75	168	1.91 (0.97-3.74)	3.56	0.059
Male gender	168	1.32 (0.59-2.95)	0.45	0.507
**Baseline BMI ≤25**	**166**	**3.69 (1.81-7.53)**	**13.12**	**<0.001**
Baseline FVC % predicted ≤65%	167	1.39 (0.71-2.71)	0.90	0.342
**Baseline DLco % predicted ≤35%**	**154**	**4.50 (2.11-9.59)**	**15.68**	**<0.001**
Baseline oxygen saturations ≤94%	162	1.63 (0.81-3.28)	1.93	0.166
Baseline PPI treatment	168	0.95 (0.49-1.85)	0.02	0.876
Baseline statin treatment	168	1.23 (0.63-2.40)	0.36	0.551
**Baseline supplementary oxygen**	**168**	**2.50 (1.09-5.76)**	**4.54**	**0.028**
Prior steroid therapy	168	0.78 (0.29-2.11)	0.24	0.628
Baseline eGFR ≤50	167	1.47 (0.66-3.27)	0.87	0.344
Baseline neutrophil : lymphocyte ratio ≥5	166	1.94 (0.87-4.32)	2.52	0.104
Baseline platelet count ≤200	167	1.51 (0.75-3.04)	1.33	0.246

Supplementary Table 4: Multivariate analysis for 12-month mortality

**Variable**	**Odds ratio (CI)**	**P value**
Baseline BMI ≤25	3.30 (1.44-7.59)	0.005
Baseline DLco % predicted ≤35	3.30 (1.44-7.59)	0.005

Supplementary Table 5: Multivariate analysis for 6-month mortality

**Variable**	**Odds ratio (CI)**	**P value**
Baseline DLco % predicted ≤35	4.34 (1.47-13.12)	0.008
Baseline eGFR ≤50	4.51 (1.39-14.57)	0.012
Baseline neutrophil : lymphocyte ratio ≥5	3.63 (1.14-11.55)	0.029

Supplementary Table 6: Multivariate analysis for 18-month mortality

**Variable**	**Odds ratio (CI)**	**P value**
Baseline BMI ≤25	2.96 (1.33-6.57)	0.008
Baseline DLco % predicted ≤35	3.83 (1.73-8.51)	0.001

Supplementary Table 7: Multivariate analysis for 12-month mortality including only antifibrotic-treated patients

**Variable**	**Odds ratio (CI)**	**P value**
Baseline BMI ≤25	14.40 (3.68-56.34)	<0.001
Baseline platelet count ≤200	4.09 (1.13-14.77)	0.031
Discontinuation of an antifibrotic within 3 months	6.26 (1.54-25.52)	0.010

Univariate and multivariate analysis for PFS

Supplementary Table 8: Univariate analysis for 12-months PFS

**Variable**	**N**	**Odds ratio (CI)**	**Likelihood ratio**	**P value**
**Age ≥75**	**168**	**0.48 (0.26-0.90)**	**5.32**	**0.022**
Male gender	168	0.75 (0.37-1.54)	0.61	0.435
**Baseline BMI ≤25**	**166**	**0.40 (0.20-0.79)**	**7.23**	**0.008**
Baseline FVC % predicted ≤65%	167	1.41 (0.76-2.62)	1.17	0.279
Baseline DLco % predicted ≤35%	154	0.56 (0.28-1.11)	2.79	0.096
Baseline oxygen saturations ≤94%	162	0.71 (0.38-1.33)	1.16	0.283
Baseline PPI treatment	168	0.59 (0.32-1.10)	2.82	0.094
Baseline statin treatment	168	1.08 (0.59-1.99_	0.07	0.798
Baseline supplementary oxygen	168	0.44 (0.18-1.06)	3.64	0.061
Prior steroid therapy	168	1.91 (0.79-4.58)	2.11	0.145
Baseline eGFR ≤50	167	0.96 (0.45-2.05)	0.01	0.917
Baseline neutrophil : lymphocyte ratio ≥5	166	0.70 (0.32-1.54)	0.81	0.370
Baseline platelet count ≤200	167	0.83 (0.43-1.59)	0.32	0.573

Supplementary Table 9: Multivariate analysis for 12-month PFS

**Variable**	**Odds ratio (CI)**	**P value**
Age ≥75	2.12 (1.08-4.14)	0.029

Supplementary Table 10: 12-month PFS with antifibrotic treatment, adjusting for age >75

**Variable**	**Odds ratio (CI)**	**P value**
Age ≥75	1.79 (0.93-3.44)	0.081
Antifibrotic treatment	0.60 (0.31-1.18)	0.139

Multivariate analysis for 6-month PFS
No significant results.

Multivariate analysis for 18-month PFS
No significant results.

Antifibrotic discontinuation univariate and multivariate analysis

Supplementary Table 11: Univariate analysis for antifibrotic discontinuation by 12 months

Variable	N	Odds ratio (CI)	Likelihood ratio	P value
**Age ≥75**	**104**	**2.35 (1.02-5.38)**	**4.13**	**0.042**
**Female sex**	**104**	**3.07 (1.20-7.82)**	**5.75**	**0.016**
**Baseline BMI ≤25**	**102**	**4.33 (1.66-11.28)**	**9.73**	**0.002**
Baseline supplementary oxygen	104	1.80 (0.67-4.80)	1.38	0.239
Baseline DLco % predicted ≤35	96	2.07 (0.87-4.94)	2.74	0.097
Baseline FVC % predicted ≤65	103	0.75 (0.34-1.63)	0.54	0.463
Baseline oxygen saturations ≤94	101	1.50 (0.68-3.32)	1.01	0.316
Baseline eGFR ≤50	104	1.59 (0.59-4.31)	0.82	0.362
Baseline platelet count ≤200	104	1.78 (0.77-4.13)	1.82	0.176

Supplementary Table 12: Multivariate analysis for antifibrotic discontinuation by 12 months

Variable	Odds ratio (CI)	P value
Age ≥75	3.17 (1.16-8.68)	0.025
Female sex	4.57 (1.40-14.88)	0.012
Baseline BMI ≤25	3.74 (1.22-11.53)	0.021
